# Regulation Effect of *Toxocara canis* and Anthelmintics on Intestinal Microbiota Diversity and Composition in Dog

**DOI:** 10.3390/microorganisms12102037

**Published:** 2024-10-09

**Authors:** Na Wang, Soben Sieng, Ping Chen, Tian Liang, Jingyun Xu, Qian Han

**Affiliations:** 1 Laboratory of Tropical Veterinary Medicine and Vector Biology, Hainan Province Key Laboratory of One Health, School of Life and Health Sciences, Collaborative Innovation Center of One Health, Hainan University, Haikou 570228, China; wangna@hainanu.edu.cn (N.W.);; 2 Faculty of Veterinary Medicine, Royal University of Agriculture, Dongkor District, Phnom Penh 120501, Cambodia

**Keywords:** *Toxocara canis*, anthelmintics, intestinal microbiota

## Abstract

*Toxocara canis* is an intestinal roundworm that can cause serious zoonotic parasitic diseases. Drontal Plus^®^ Tasty (Dog) is a kind of commercial drug used to treat *T. canis* infection. Febantel, Praziquantel, and Pyrantel pamoate (PP) are its main component. However, there are few studies investigating the impact of Drontal Plus^®^ Tasty (Dog) and its primary ingredients on the intestinal microbiota of dogs. In this study, we first collected the intestinal content samples of the dogs which administrated with anthelmintics or saline by sterile catheters, then used 16S rRNA high-throughput sequencing technology combined with a variety of bioinformatic analysis methods to analyze the effect of anthelmintics on intestinal microbiota. First, the results of the α and β diversity analysis showed that the abundance and diversity of intestinal microbiota decreased with *T. canis* infection, and increased after anthelmintic treatment. Then, we found the dominant species (the value of relative abundance > 0.05) was both 28 on phylum and genus levels, besides the most dominant species was Bacillota on phylum level and *Segatella* and *Clostridium_sensu_stricto* were most dominant on genus level. Futher analyzing the differences in microbiotal composition on phylum level, we found that Drontal Plus^®^ Tasty treatment could significantly increase the proportion of Bacillota, while Febantel, Praziquantel, or PP could induce the significantly changes of Bacillota and Bacteroidota. In addition, by analyzing the differences in microbiotal composition on genus level, we found that anthelmintic could significantly decreased the relative abundance of *Clostridium_sensu_stricto* and significantly increased the abundance of *Segatella*. However, Drontal Plus^®^ Tasty had no regulatory effect on the abundance of *Segatella*. In short, these finding showed that various anthelmintics all have significant effects for changing the abundance and diversity of host intestinal microbiota.

## 1. Introduction

*Toxocara canis* is of great significance in veterinary medicine and public health, and its adults reside in the small intestine of canids [1]. *T. canis* initiates its life cycle when eggs are accidentally ingested by a host. The larvae hatch in the host’s intestine, migrate to tissues, particularly the liver and lungs for parasitism. In the definitive host, dogs, *T. canis* larvae traverse the alveoli, bronchioles, and bronchi, ultimately being swallowed into the stomach through the throat, reaching the small intestine where they further develop into adults. The parasitism and migration of *T. canis* inflict severe harm on the host, including various intestinal disorders, organ damage, and inflammatory reactions. Toxocariasis is a neglected zoonotic parasitic disease worldwide, especially in low- and middle-income countries and areas with poor health conditions [2,3]. The intestinal microbiome is normally constituted of bacteria, viruses and some eukaryotic organisms that reside in the gastrointestinal (GI) tract, and form the symbiotic fashion with the hosts [4]. The differentiation of microbial communities along the GI tract is a reflection of the microenvironment and physiological functions. The gut microbiome contributes to host metabolism and development of the immune system, protects against pathogens, and along with these functions, it possibly directly or indirectly affects most physiologic functions of its host [5].

Interestingly, some studies in humans have found increased intestinal microbial diversity in individuals infected with gut helminths [6,7,8] or protozoa [9], and the changes in diversity following anthelmintic treatment were also detected [10]. A notable reduction in the abundance of Clostridia species, alongside a general decline in overall bacterial diversity, was observed among children which co-infection with *Trichuris trichiura* and *Ascaris lumbricoides* [11]. Conversely, Lee et al., reported a significant increase in bacterial diversity among individuals living in rural Malaysia which infected with any helminth species [6]. *Necator americanus* infection would also increased the intestinal bacterial richness of the host [12]. In addition, administering *Lactobacilli taiwanensis* to BALB/c mice significantly promoted the establishment of *Heligmosomoides polygyrus bakeri* [13]. *Lactobacillus casei* has been shown to decrease adult worm burdens of *T. spiralis* [14], and Bifdobacterium animalis strain 04450B provided protection against *Strongyloides venezuelensis* infection [15]. On the other hand, post-albendazole treatment resulted in significant variations in the microbiota [16]. Ivermectin induced minor and transient alterations in the intestinal microbiota [17]. Pierre et al. emphasized that when albendazole-ivermectin was used to treat *T. trichiura* and hookworm infections, the intestinal microbiota emerged as a key factor that determined the success of treatment [18].

Studies have shown that the gastrointestinal diseases and inflammation response in dogs are also closely related to the disturbance of intestinal microbiota [19,20,21]. Šlapeta et al. observed a statistically significant difference in the bacterial genera between the Giardia-positive and Giardia-negative dogs. Conversely, no such significant difference was observed between dogs that tested positive or negative for Ancylostoma infection [22]. In our previous study, we found that *T. canis* infection could increased the abundance of Firmicutes and Planctomycetes in dog’s intestinal microbiota. Further analysis of the differentially expressed bacteria on genus level, it was found that some of the pathogenic bacteria, such as *Clostridium_sensu_stricto* and *Staphylococcus* spp. were also increased [23]. In addition, antibiotic treatment also affected the composition and diversity of intestinal microbiota in dogs. Igarashi et al. [24] discovered a marked decrease in the intestinal bacterial diversity index of dogs when subjected to a twice-daily injection dose of 12.5 mg/kg of methnidazole. Conversely, administering a once-daily dose of 1.0 mg/kg of prednisolone did not elicit any significant changes in the abundance or composition of the microbiota. These results indicated that variations in drug type and dosage could exert distinct regulatory influences on the intestinal microbiota of dogs.

In general, helminth infection could cause changes in the host’s intestinal microbiota, and drugs also have regulatory effects on the intestinal microbiota. Drontal Plus^®^ Tasty is currently the most widely used commercial anthelmintic. However, the effects of Drontal Plus^®^ Tasty and its main component Febantel, Praziquantel, and Pyrantel pamoate (PP) on the host’s gut microbiota are not well understood. Therefore, this study will focus on analyzing the changes in composition and diversity of the intestinal microbiota of dogs which infected with *T. canis* after anthelmintic treatment. The primary objective is to evaluate the potential effects of anthelmintic on the homeostasis of intestinal microbiota, thereby providing a basis for standardizing medication guidelines.

## 2. Materials and Methods

### 2.1. Animals and Ethics Statement

A total of 18 female chinese rural dogs less than six months of age which kept together in the same environment were used in this experiment. The dogs were housed in individual cages with free access to a standard diet (200 g same commercial dog food per dog per day and free drinking water) and acclimatized to the experimental environment (26–28 °C, 80–85% humidity). During the experiment, we monitored the health of the dogs through observing their activity level, appetite and water intake, excretion, stool status and weight change to ensure that the experimental dogs in good state. The utilization of dogs underwent health checks and behavioral evaluation sessment, and received official approval from the Hainan University Institutional Animal Care and Use Committee (HNUAUCC-2023-00201).

### 2.2. Experiment Grouping and Sampling

Stool samples were collected from ground of each dog prior to feeding anthelmintic, and feces were tested for infection with helminth eggs by water wash precipitation method. After conducting fecal examinations, 15 dogs testing positive for *T. canis* infection and 3 dogs testing negative (CI group) were selected. And there were no other types of helminth infection. Among the positive dogs, 3 were administered Drontal Plus^®^ Tasty at a dosage of one tablet per 10 kg of body weight in accordance with the manufacturer’s recommendations (TI group). The remaining 9 positive dogs were then randomly divided into three separate groups. Each group received treatment according to the following dosages per 10 kg of body weight: Febantel 15 mg (Febantel group), Praziquantel 5 mg (Praziquantel group), and Pyrantel pamoate 14.4 mg (PP group). Another 3 positive dogs were given normal saline as control (II group). On the 7th day after anthelmintic treatment, the dogs were subjected to anesthesia, and within a sterile operating environment, sterile catheters were used to procure a minimum of 2 mg of intestinal content samples from the rectal ampullae of the six groups. Throughout the sampling procedure, we ensure meticulous adherence to sterile practices, thereby preventing any potential contamination of the samples. After collection, each sample was put into an appropriate sterile tube, which was then snap-frozen in a liquid nitrogen tank. Besides, prior to collecting samples of intestinal contents, we also collected each dog’s naturally excreted feces and examined them for parasites, and no *T. canis* eggs or worms were detected in the feces. The experimental groups were shown in Supplementary Table S1.

### 2.3. 16S rRNA High-Throughput Sequencing and Bioinformatics Analysis

The sequencing and analysis are performed according to a previously published work [23]. First, the sample DNA was extracted used the QIAamp DNA Stool Mini Kit (Qiagen, Hilden, Germany) and the Qubit Fluorometer Kit (Life Technologies, Carlsbad, CA, USA) was used to measure the DNA concentration. PCR amplification was performed using a PCR reaction system configured with 30 ng of DNA samples and corresponding fusion primers. The PCR amplification products were purified using Agencourt AMPure XP magnetic beads and dissolved in Elution Buffer for subsequent library preparation. The fragment size range and concentration of the library were detected using an Agilent 2100 Bioanalyzer. Qualified libraries were sequenced based on the size of the inserted fragments. The raw sequencing data were filtered, and the remaining high-quality clean data were used for subsequent analysis. To analyze the taxonomic composition of each sample, the obtained sequences were grouped into operational taxonomic units (OTUs) using a 97% sequence identity threshold. Subsequently, the Mothur algorithm was used to assign taxonomic classifications to each OTU sequence, leveraging the SILVA 16S rRNA database (Version 128). To assess the α and β diversities, the Quantitative Insights Into Microbial Ecology (QIIME) database was employed, α diversity measures can be seen as a summary statistic of a single population (within-sample diversity), while β diversity measures are estimates of similarity or dissimilarity between populations (between samples). The β diversity index compares the differences in the quantity and distribution of each species between two communities in a matrix format. A lower β diversity indicates less variability within the community. Here we choice coverage, simpson index to measure α diversity and weighted unifrac index was used to measure the β diversity. For identifying taxa that exhibited variations between groups, the linear discriminant analysis effect size (LEfSe) method was implemented. Additionally, LDA (linear discriminant analysis) was utilized to evaluate the impact of specific taxa. The Jensen-Shannon Distance and Partitioning Around Medoids (PAM) methods were applied to calculate distances, based on the relative abundance of taxa, focusing on the phylum or genus level. The optimal number of clusters, denoted as the K-value, was determined using the Calinski-Harabasz index. Finally, the outcomes of the clustering analyses were visualized using between-class analysis or principal coordinates analysis.

### 2.4. Statistical Analysis

A one-way analysis of variance (ANOVA), Wilcox Test, and Kruskal Test were used for comparisons between groups. The threshold for determining significant differences was set at a *p* value less than 0.05.

## 3. Results

### 3.1. 16S rRNA High-Throughput Sequencing Result

**Figure 1:**
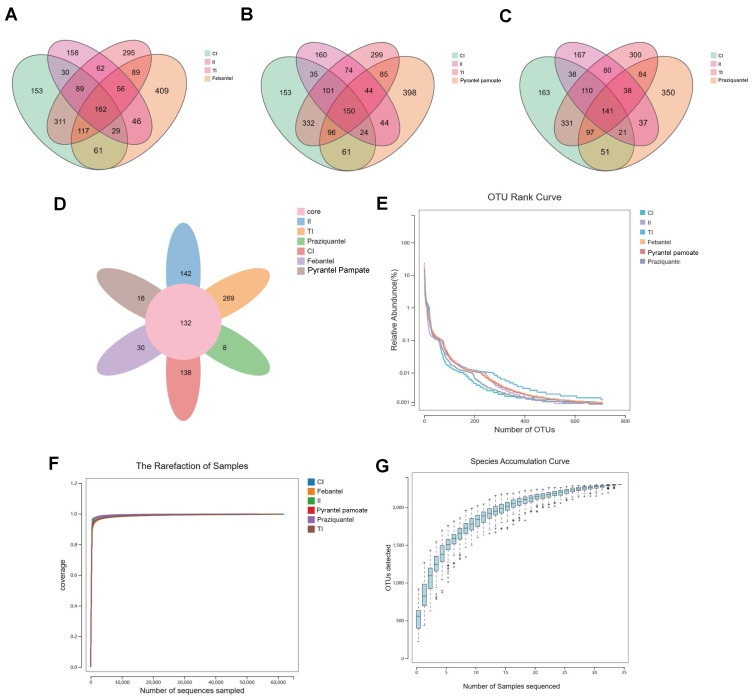
OUT statistical analysis. (**A**–**D**) Venn diagram and core-pan OUT map of the unique and shared OTUs among the six groups. (**E**) Rank-Abundance curves. (**F**) Rarefaction Curve Analysis. (**G**) Species accumulation curve. The horizontal coordinate represents the number of sequencing samples, and the vertical coordinate represents the number of OTUs. CI, intestine samples of dogs in the control group; II, intestine samples of dogs in *T. canis* infected group; TI, intestine samples of dogs infected with *T. canis* after Drontal Plus^®^ Tasty treatment group; Febantel, intestine samples of dogs infected with *T. canis* treated with Febantel; Praziquantel, intestine samples of dogs infected with *T. canis* treated with Praziquantel; PP, intestine samples of dogs infected with *T. canis* treated with Pyrantel pamoate.

The numbers in the multiple overlapping part of the Veen figure mean the number of the common OTUs among multiple groups, and the unoverlapping part is the number of OUT unique to the group. The results of Figure 1A–C showed that anthelmintic treatment significantly increased the richness of the intestinal microbiota. Meanwhile, the Core-pan OUT map indicated that the number of unique OTUs in each group was 138, 142, 269, 30, 8, and 16 in the CI, II, TI, Febantel, Praziquantel, and PP groups, respectively (Figure 1D). According to the comprehensive analysis of the results in Figure 1A–D, it was found that the effects of Febantel, Praziquantel, and PP treatment on the richness of intestinal microbiota were relatively similar and all significantly lower than CI and II group. While, Drontal Plus^®^ Tasty treatment group was significantly different, the richness of intestinal microbiota in TI group was higher than the other five groups. On the other hand, as shown in Figure 1E, the Rank-Abundance curve demonstrated that the richness and the evenness of the microbiota in the six groups were not statistically significant. The rarefaction curve of each group in this study tended to be flat, indicating that the amount of sequencing data was large enough to reflect the vast majority of microbial information in the samples of the six groups (Figure 1F). Meanwhile, the upward trend at the end of the species accumulation curve tended to flatten out, it indicated that the sample size was sufficient to reflect the species composition of the community (Figure 1G). In general, our experimental results had proved to be reliable and could reflect the real changes of intestinal microbiota.

### 3.2. Diversity Analysis

#### 3.2.1. Alpha Diversity Analysis

**Figure 2:**
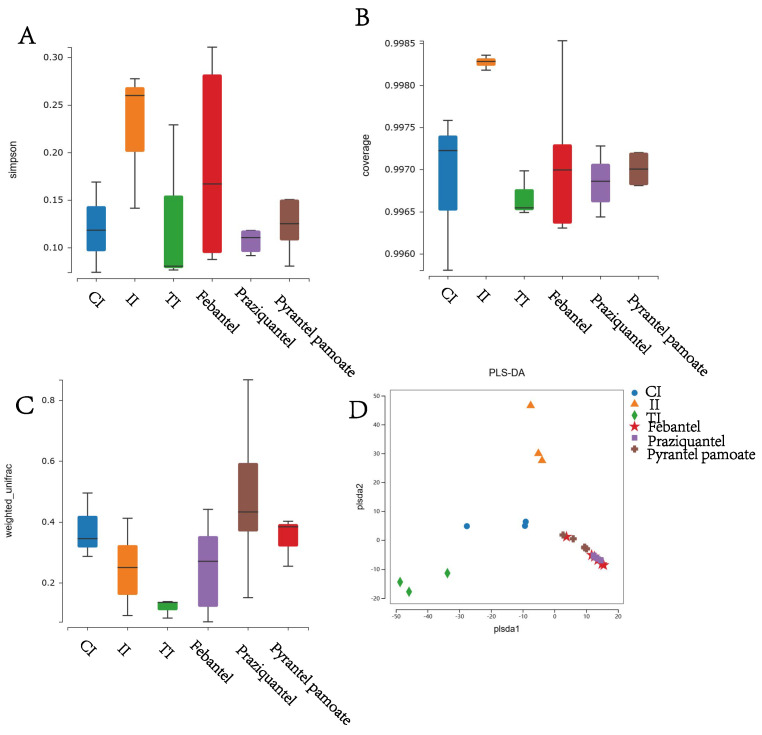
Comparison of Alpha and Beta diversity index of intestinal microbiota among the six groups. (**A**) Coverage index. (**B**) Simpson index. (**C**) Weighted unifrac. (**D**) Partial Least−Squares Discriminant Analysis (PLS−DA).

To investigate the diversity of the bacterial species in the intestine of the six groups, the α diversity was measured as shown in Figure 2A,B. In this study, we used the Coverage, Simpson index to evaluate the diversity of microbial communities. The Good’s coverage index of each group was higher than 99%, which indicated that the sequences of samples were reliable. And the Simpson index showed that the richness, diversity and evenness of intestinal microbiota of the II group was higher than the CI group, while the richness, diversity and evenness of intestinal microbiota were significantly decreased after anthelmintic treatment. Our results demonstrated that anthelmintic treatment could alter the abundance and diversity of intestinal microbiota.

#### 3.2.2. Beta Diversity Analysis

Beta diversity indicates the comparison of species presence, abundance and phylogenetic relationships among community members. In our study, the weighted unifrac index indicated that the species composition of intestinal microbiota was more stable compared the II group with the anthelmintic treatment groups. The effects of different anthelmintic treatment on intestinal microbiota were also different, but the difference was not obvious between the CI group and the anthelmintic treatment groups (Figure 2C). The result of PLS-DA analysis showed that the CI, II and TI groups formed different clusters, indicating that the sample grouping effect was well and the difference between groups was obvious. But the difference between the Febantel, Praziquantel, and PP groups were small and could not be distinguished well (Figure 2D). These results indicated that the three anthelmintic had certain similarities in their effects on intestinal microbiota.

#### 3.2.3. LEfSe Analysis

LEfSe analysis aided in determining the biomarkers that were significantly differently present among the different groups. As illustrated in Supplementary file (Figure S1), LEfSe analysis of the six groups elucidated 22 biomarkers with LDA score > 4. In the TI group, *Peptostreptococcaceae*, *Clostridioides*, *Micrococcales*, *Bacillales_Incertae Sedis XI* and *Gemella* could be used as biomarkers. In the Febantel group, *Negativicutes*, and *Anaerovibrio* could be used as biomarkers. In the Praziquantel group, *Succinivibrionaceae*, *Aeromonadales*, *Anaerobiospirillum*, and *Sutterellaceae* could be used as biomarkers. In the PP group, *Cellulosilyticum*, and *Veillonella* could be used as biomarkers. And LEfSe clustering analysis showed that there were significant differences between the six groups at different levels Supplementary file (Figure S2).

### 3.3. Species Composition and Difference Analysis

#### 3.3.1. Species Composition and Difference Analysis on Phylum Level

**Figure 3:**
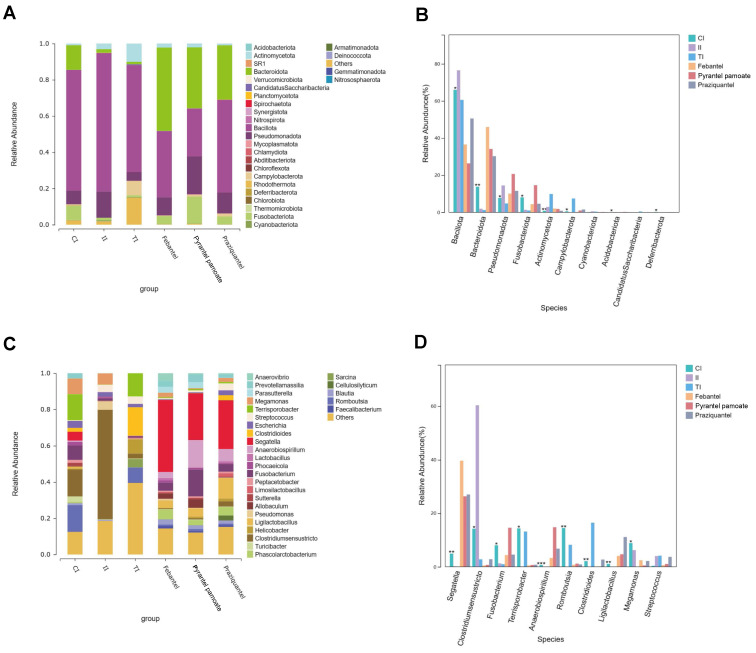
Difference in species composition of microbiota of the six groups on Phylum and genus levels. The horizontal coordinate is the sample name and the vertical coordinate is the relative abundance of the species annotated. Species not annotated at this taxonomic level and whose abundance was less than 0.5% of the sample were combined as “Others” (**A**,**C**). The top ten species on phylum level in the six groups were showed in (**B**). And the top ten species on genus level in the six groups were showed in (**D**). The significance of the test of difference were marked with * at the top of the bar graph if available, or not marked if not. * indicated the *p* value ≤ 0.05; ** indicated the *p* value ≤ 0.01; *** indicated the *p* value ≤ 0.001.

As shown in Figure 3A, the species composition histogram could explain the dominant species and their relative abundance of each group. The dominant species (the value of relative abundance > 0.05) in the intestinal microbiota of the CI group were from phyla Bacillota, Bacteriodota, Pseudomonadota, and Fusobacteriota. In the II group, the dominant species were from phyla Bacillota, and Pseudomonadota, while in the TI group, the dominant species were from phyla Bacillota, Actinobacteria, and Campylobacterota. In the Febantel group, the dominant species were from phyla Bacteriodota, Bacillota, andPseudomonadota. In the Praziquantel group, the dominant species were from phyla Bacteriodota, Bacillota, Pseudomonadota and Fusobacteriota. In the PP group, the dominant species were from phyla Bacillota, Bacteriodota, and Pseudomonadota. In short, Bacillota was the dominant phylum in all groups. However, after different treatments, the abundance of Bacillota in each group changed significantly. The differences in the abundance of intestinal microbiota on phylum level among all groups were shown in Figure 3B. Compared with the CI group, the community abundance of Bacillota in the II group was significantly increased, and significantly decreased after anthelmintic treatment (*p* ≤ 0.05). In addition, we found that Bacteriodota was significantly decreased with *T. canis* infection, and significantly increased after febantel, praziquantel, or PP treatment, while Drontal Plus^®^ Tasty treatment has no regulatory effect on the reduction of Bacteriodota caused by *T. canis* parasitism.

#### 3.3.2. Species Composition and Difference Analysis on Genus Level

Through comparing the microbiota composition changes in all groups on genus level (Figure 3C), illustrating that the dominant genera (the value of relative abundance ≥ 0.05) in the CI group were *Romboutsia*, *Clostridium_sensu_stricto*, *Megamonas*, *Terrisporobacter*, and *Fusobacterium*. The dominant genera in the II group were *Clostridium_sensu_stricto* and *Megamonas*. While in the TI group, the dominant genera were *Romboutsia*, *Clostridioides*, *Helicobacter*, and *Terrisporobacter*. In the Febantel group, the dominant genera were *Segatella*, and *Phascolarctobacterium*. In the Praziquantel group, the dominant genera were *Segatella*, *Anaerobiospirillum*, and *Fusobacterium*. In the PP group, the dominant genera were *Segatella*, *Anaerobiospirillum*, and *Ligilactobacillus*. The differences in the abundance of intestinal microbiota on genus level among all groups were shown in Figure 3D. The relative abundance of *Clostridium_sensu_stricto* was increased after infected with *T. canis*, and its value was decreased after treatment with anthelmintic. And the relative abundance of *Clostridium_sensu_stricto* in the febantel and PP treatment groups were lower than that in the Drontal Plus^®^ Tasty and praziquantel treatment groups. While, the relative abundance of *Segatella* was decreased after infection, and its value was significantly increased after treatment with febantel, praziquantel, and PP. However, Drontal Plus^®^ Tasty had no regulatory effect on the decrease of the abundance of *Segatella* caused by *T. canis* infection.

### 3.4. Interconnections between Major Microbiota

**Figure 4:**
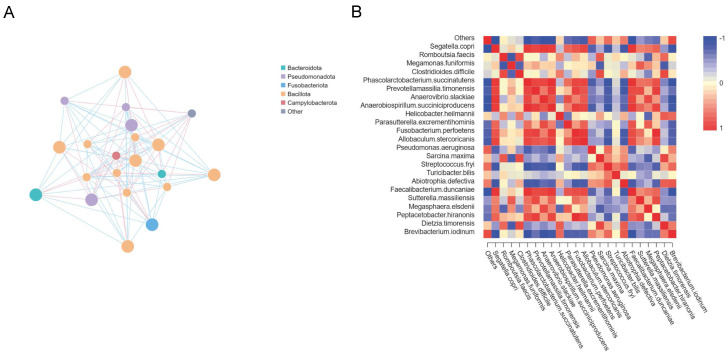
Correlation between microbiota. Correlation networks of microbiome on phylum showed in (**A**), each node in the graph represents a species, the larger the area of the point, it means that the average relative abundance of the species is higher; the species are connected to the species using straight lines, the pink color indicates a positive correlation, the blue color indicates a negative correlation, and the thickness of the lines indicate the correlation magnitude (the figure only shows the correlation coefficient between the species is more than 0.2). Heat map of correlation coefficient of microbiome on species was showed in (**B**). Darker colors indicating stronger correlation between species.

Construction networks revealed that the phylum Bacillota showed more number and larger area of the points; it meant that the relative abundance of the species is higher. At the same time, there was an interaction between Bacillota, Bacteroidetes, Fusobacteriota, Pseudomonadota, and Campylobacterota (Figure 4A). Besides, we performed heat map of correlation analysis to illustrate the correlation between species that differ at all taxonomic levels (Figure 4B).

## 4. Discussion

The intricate and multifaceted intestinal microbiota residing within animals engages in a harmonious symbiosis with host. It’s well known that intestinal microbiota of dogs play important functions in various life processes such as metabolism, immunity and neurobehavior, including degrading indigestible carbohydrates and producing metabolic products such as short chain fatty acid (SCFAs), which can provide energy for colon cells and play a key role in maintaining the integrity of the intestinal epithelial barrier [25,26,27]. Since *T. canis* resides in the intestine of dogs, the change of intestinal microbiota caused by *T. canis* infection and the mechanism of the intestinal microbiota interaction with the mucosal immune cells have gained increased intention in the last few decades. Our previous study has confirmed that *T. canis* infection could elicit significant alterations in both the abundance and diversity of the intestinal microbiota in dogs [23]. Furthermore, apart from the well-known influence of antibiotics, a few studies have confirmed that anthelmintics therapy might alter the susceptibility of hosts to parasitic infections through parasite-microbe interactions [28,29]. However, there are relatively few studies on the effects of anthelmintic on the intestinal microbiota composition of dogs.

Drontal Plus^®^ Tasty is a common commercial anthelmintic for dogs and cats, which is used to treat tapeworm, roundworm, hookworm and whipworm infections. The main ingredients of Drontal Plus^®^ Tasty (Dog) are Febantel, Praziquantel, and Pyrantel pamoate (PP). In order to make the experimental results more realistic, we selected dogs infected with *T. canis* for Drontal Plus^®^ Tasty (Dog), Febantel, Praziquantel, and PP treatment separately, and then used 16S rRNA gene amplicon sequencing technology to explore the changes in species composition, relative abundance, and diversity of the intestinal microbiota. More important, in order to eliminate the influence of diet, age and other factors on the accuracy of experimental results, we ensured a rigorous standard of uniformity in the selection of dogs, their feeding protocols, as well as the location and methodology utilized for sampling.

Currently, there is a relatively more research investigating the impact of anthelmintics on the intestinal microbiota of equine animals. Multiple studies have shown that parasitic infections are associated with an increase in microbial richness and diversity, while the administrate of anthelmintics is linked to a decrease in α diversity within the intestinal microbial community [30,31,32]. Walshe et al. [30] found that both α diversity and β diversity decreased in horse fecal samples at seven days after treatment with fenbendazole and moxidectin, which attributed to the degradation of parasites. And no differences were observed between the two anthelmintics treatment groups. Kunz et al. [31] demonstrated that moxidectin and praziquantel reduced microbial diversity, but β diversity remained unchanged when using weighted UniFrac method. Additionally, significant differences were found in the relative abundance of 21 taxonomic groups. Similarly, Daniels et al. [32] reported that moxidectin did not significantly alter bacterial diversity, but there were 13 distinct OTUs that differed between the treatment and control groups. Through analyzing the changes in intestinal microbiota diversity before and after anthelmintics treatment in dogs, we found that α diversity increased following infection with *T. canis* and decreased after treatment with different anthelmintics. We believed this was also related to the expulsion or degradation of *T. canis*, though the differences between groups were not significant, which was attribute to significant inter-individual variation. Although we tried to avoid the influence of breed, diet and environment on intestinal microbiota during breeding, there were still significant individual differences in microbiota. However, β diversity significantly decreased after *T. canis* infection but significantly increased after anthelmintics treatment, suggesting that the fluctuations in intestinal microbiota after *T. canis* infection were relatively smaller, whereas anthelmintics treatment caused relatively larger fluctuations in the composition of microbiota.

On phylum level, Bacillota (Formerly known as Firmicutes), Bacteroidota, Fusobacteriota, and Pseudomonadota are the dominant microbiota in all groups, this is consistent with other research findings [33]. A previous study demonstrated that all butyrate-producing bacteria belong to the Firmicutes [34]. Besides, butyrate is one of the most important fatty acids associated with anti-inflammatory activity [35]. In our study, the ratio of Bacillota was increased after dogs being infected with *T. canis*, and decreased after being treated with anthelmintics. At the same time, Febantel, Praziquantel, andPP treatment caused the greater degree of Bacillota reduction than Drontal Plus^®^ Tasty treatment group. We hypothesized that *T. canis* could induce a significant increase in the abundance of Bacillota, thereby inducing the anti-inflammatory environment of the host intestine. And anthelmintics could regulate the intestinal anti-inflammatory environment by reducing the abundance of Bacillota. On the other hand, Febantel, Praziquantel, or PP treatment may cause intestinal fatty acid metabolism disorders due to a significant decrease in the proportion of Bacillota in the intestine, which has a certain negative impact on function of intestinal microbiota.

Meanwhile, in the Febantel, Praziquantel, and PP treatment groups, Bacteroidetes was also a kind of dominant microbiota. Some studies have shown that Bacteroidetes was decreased in patients with Blastocystis, *Entamoeba histolytica*, Toxoplasma infections [36]. Bacteroidetes also participates in regulating inflammatory processes. Our study revealed that *T. canis* infection significantly diminished the abundance of Bacteroidetes in the intestinal microbiota. Notably, the administration of Drontal Plus^®^ Tasty did not exert a notable regulatory effect on reversing the reduction of Bacteroidetes induced by *T. canis* infection. In contrast, treatment with Febantel, Praziquantel, or PP individually led to a significant increase in the proportion of Bacteroidetes, with the values observed being significantly elevated compared to that in the control group (CI group). This indicated that the different anthelminth might exerted different regulatory effects on host’s intestinal microbiota.

Additionally, Febantel, Praziquantel, or PP might exerted their function of regulating intestinal metabolism mainly by regulating the Bacillote/Bacteroidetes ratio, thus affecting the infectivity of intestinal parasites. Yang et al. [10] found that *Enterobius vermicularis* infection in children was associated with a decrease in Fusobacteria phylum. Mebendazole treatment of *E. vermicularis* infection in primary school children in Taiwan province of China caused a relative decrease in Fusobacteriota. Fusobacteriota may be involved in homeostasis maintenance and metabolic function of intestinal microbiota. In our study, we found that the proportion of Fusobacteriota in the Drontal Plus^®^ Tasty treatment group is significantly reduced, which is not only much lower than the control group (CI group), but even lower than the *T. canis* infection group, which is consistent with the previous experimental results [10]. However, the difference is that the proportion of Fusobacteriota in Febantel, Praziquantel, and PP treatment groups were increased, and the proportion of Fusobacteriota in Praziquantel treatment was even higher than that in the control group (CI group). In general, different anthelmintics have different effects on the intestinal microbiota. On one hand, they can regulate the intestinal microbiota caused by parasitic infections, on the other hand, they can also lead to the intestinal microbiota imbalance.

Through conducting a comparative analysis of key species differences on genus level, we observed that *Clostridium_sensu_stricto* was increased after *T. canis* infection and decreased after anthelmintic treatment. Previous studies have confirmed that *Clostridium_sensu_stricto* may be involved in certain metabolic processes in the gut and considered as a well-known pro-inflammatory and a colitis-inducing bacterium [37]. *Romboutsia*, and *Terrisporobacter* significantly decreased after *T. canis* infection and increased after anthelmintic treatment. Moreover, the effect of Drontal Plus^®^ Tasty treatment on the microbiota abundance was significantly greater than that of Febantel, Praziquantel, and PP treatment groups. *Romboutsia* can convert macromolecular carbohydrates to short-chain fatty acids (SCFAs) such as butyric acid, thus maintaining the stability of intestinal microbiota [38]. *Terrisporobacter* has been linked to short-chain fatty acid and oxidative stress in animal studies [39]. Overall, anthelmintic treatment could relieve intestinal inflammatory response and metabolic disorders by increasing short-chain fatty acid.

Furthermore, *Segatella*, *Fusobacterium*, *Anaerobiospirillun*, and *Ligilactobacillus* were significantly decreased after *T. canis* infection and Drontal Plus^®^ Tasty treatment, while Febantel, Praziquantel, or PP treatment could significantly increase the abundance of these microorganisms, and the abundance were even higher than the control group. *Segatella* was strong association with glucose homeostasis and host metabolism, and play a potential anti-inflammatory role [40]. Fusobacterium may regulate the intestinal environment by producing specific metabolites, such as short-chain fatty acids. In addition, *Fusobacterium* may also form a symbiotic relationship with other gut microbes to jointly resist the invasion of harmful microorganisms [41]. In addition to participating in the absorption and metabolism of nutrients, *Anaerobiospirillun* also has certain immunomodulatory effects [42]. *Lactobacillus* has ability to inhibit pathogenic bacteria, maintain the balance of intestinal microbiota, and inhibit the production of pro-inflammatory factors and play an anti-inflammatory role [43]. In short, anthelmintic can affect the homeostasis of the intestinal environment by regulating the abundance of bacteria involved in intestinal metabolism and inflammation, but different kinds of anthelmintics play different regulatory functions on different bacterial genus. We speculated that different kinds of anthelmintics may have different anthelmintics mechanisms. While they played the function of anthelmintics, they might also play a bactericidal effect on different microorganism in the intestine, so the composition of microbiota would change significantly. However, the mechanism of the different effects of different anthelmintic on intestinal microbiota is still unclear and needs to be further explored.

In addition, after Drontal Plus^®^ Tasty treatment, *Megamonas* was also almost disappeared. And the abundance of *Megamonas* in the Febantel, Praziquantel, and PP treatment groups were also significantly lower than that in the control group (CI group). Members of *Magamonas* are known to produce acetic and propionic acids, which have been discovered to be substrates for the formation of lipogenesis and cholesterol in rodents [44], lipogenesis and cholesterol accumulation may relate to liver metabolic function. We speculated that anthelmintic treatment might affect lipogenesis and cholesterol accumulation through reducing the ratio of *Magamonas*, thus leading to abnormal liver function. Moreover, the damage of *Magamonas* to the liver may be more obvious, so attention should be paid to dose control when administering medication.

## 5. Conclusions

Our study evaluated the changes in the intestinal microbiota structure of dogs with *T. canis* infection and anthelmintic treatment through various bioinformatic analyses. The results of the differentially expressed bacteria analysis on phylum and genus levels showed that anthelmintic treatment could regulate the richness and diversity of intestinal microbiota. While, various anthelmintic exerted distinct regulatory effects on diverse intestinal microbe. More important, anthelmintic treatment might cause intestinal microbiota dysfunction to a certain extent. Thus, it is critically important to pay close attention to the possible adverse effects that anthelmintic may exert on the host. During the administration of anthelmintics, it is imperative to meticulously monitor the dosage and duration of treatment, as variations in these factors can exert diverse impacts on the host’s intestinal microbiota. Furthermore, timely attention should be given to the fluctuations in various physiological parameters among dogs throughout the medication period, to avert the occurrence of severe side effects.

## Data Availability

The datasets used or analysed during the current study are available from the corresponding author on reasonable request.

## Supplementary Materials

The following supporting information can be downloaded at: https://www.mdpi.com/article/10.3390/microorganisms12102037/s1, Figure S1: LDA value distribution histogram; Figure S2: The cladogram of intestinal microbial flora in the six groups. Table S1: Description of treatment for each group.
