# Supplementary material for: Regulation Effect of Toxocara canis and Anthelmintics on Intestinal Microbiota Diversity and Composition in Dog
Source: Microorganisms. 2024 Oct 9;12(10):2037. doi: 10.3390/microorganisms12102037 (PMC11510115; doi:10.3390/microorganisms12102037)
Supplement: Supplementary file 1 [file microorganisms-12-02037-s001.zip › Figure S2.pdf]

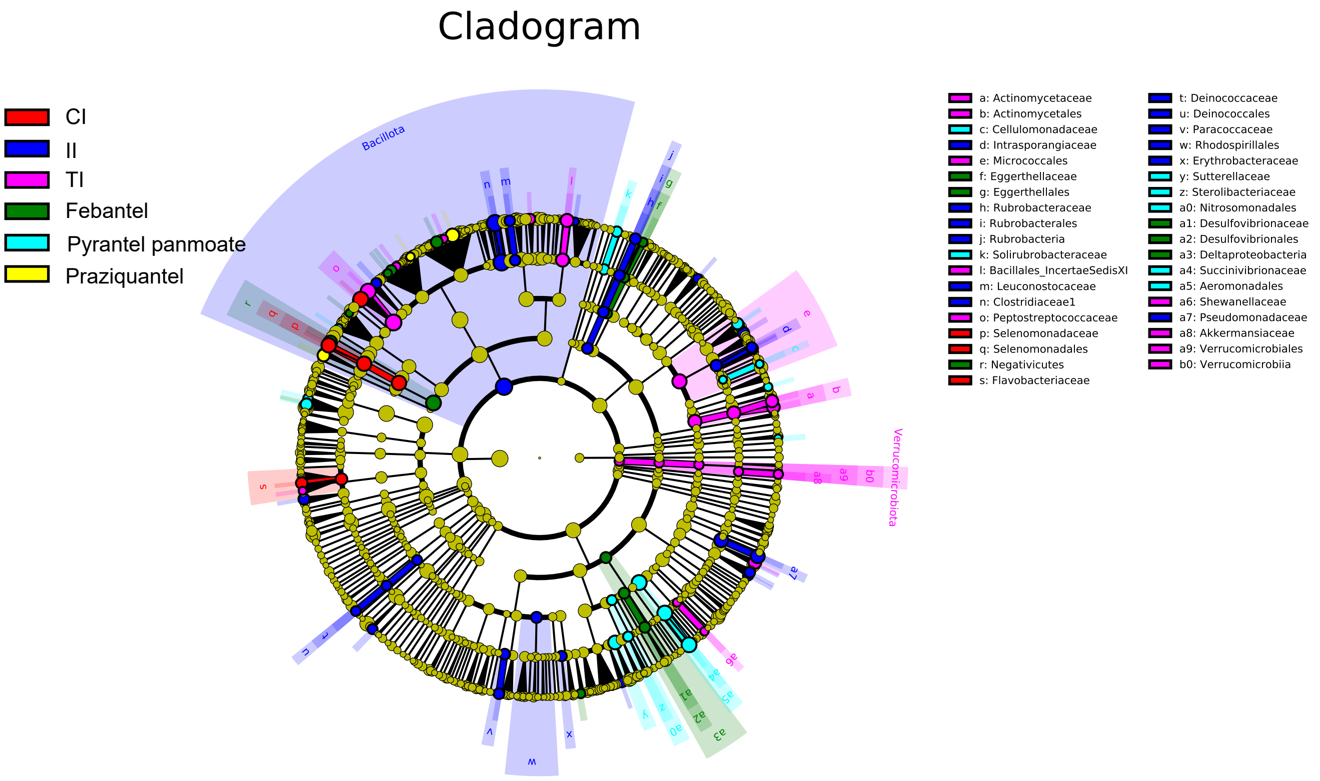

Cladogram
CI
II
TI
Febantel
Pyrantel panmoate
Praziquantel
Bacillota
Verrucomicrobiota
a: Actinomycetaceae
b: Actinomycetales
c: Cellulomonadaceae
d: Intrasporangiaceae
e: Micrococcales
f: Eggerthellaceae
g: Eggerthellales
h: Rubrobacteraceae
i: Rubrobacterales
j: Rubrobacteria
k: Solirubrobacteraceae
l: Bacillales_IncertaeSedisXI
m: Leuconostocaceae
n: Clostridiaceae1
o: Peptostreptococcaceae
p: Selenomonadaceae
q: Selenomonadales
r: Negativicutes
s: Flavobacteriaceae
t: Deinococcaceae
u: Deinococcales
v: Paracoccaceae
w: Rhodospirillales
x: Erythrobacteraceae
y: Sutterellaceae
z: Sterolibacteriaceae
a0: Nitrosomonadales
a1: Desulfovibrionaceae
a2: Desulfovibrionales
a3: Deltaproteobacteria
a4: Succinivibrionaceae
a5: Aeromonadales
a6: Shewanellaceae
a7: Pseudomonadaceae
a8: Akkermansiaceae
a9: Verrucomicrobiales
b0: Verrucomicrobiia
