# Supplementary material for: Regulation Effect of Toxocara canis and Anthelmintics on Intestinal Microbiota Diversity and Composition in Dog
Source: Microorganisms. 2024 Oct 9;12(10):2037. doi: 10.3390/microorganisms12102037 (PMC11510115; doi:10.3390/microorganisms12102037)
Supplement: Supplementary file 1 [file microorganisms-12-02037-s001.zip › Table S1.pdf]

Name	Description
CI group	Intestinal content samples of negative infected dogs
II group	Intestinal content samples of positive infected dogs which administered normal saline
TI group	Intestinal content samples of positive infected dogs which administered Drontal Plus® Tasty
Febantel group	Intestinal content samples of positive infected dogs which administered febantel
Praziquantel group	Intestinal content samples of positive infected dogs which administered praziquantel
Pyrantel pamoate group	Intestinal content samples of positive infected dogs which administered pyrantel pamoate
