# Supplementary figures and images for: Regulation Effect of Toxocara canis and Anthelmintics on Intestinal Microbiota Diversity and Composition in Dog
Source: Microorganisms. 2024 Oct 9;12(10):2037. doi: 10.3390/microorganisms12102037 (PMC11510115; doi:10.3390/microorganisms12102037)

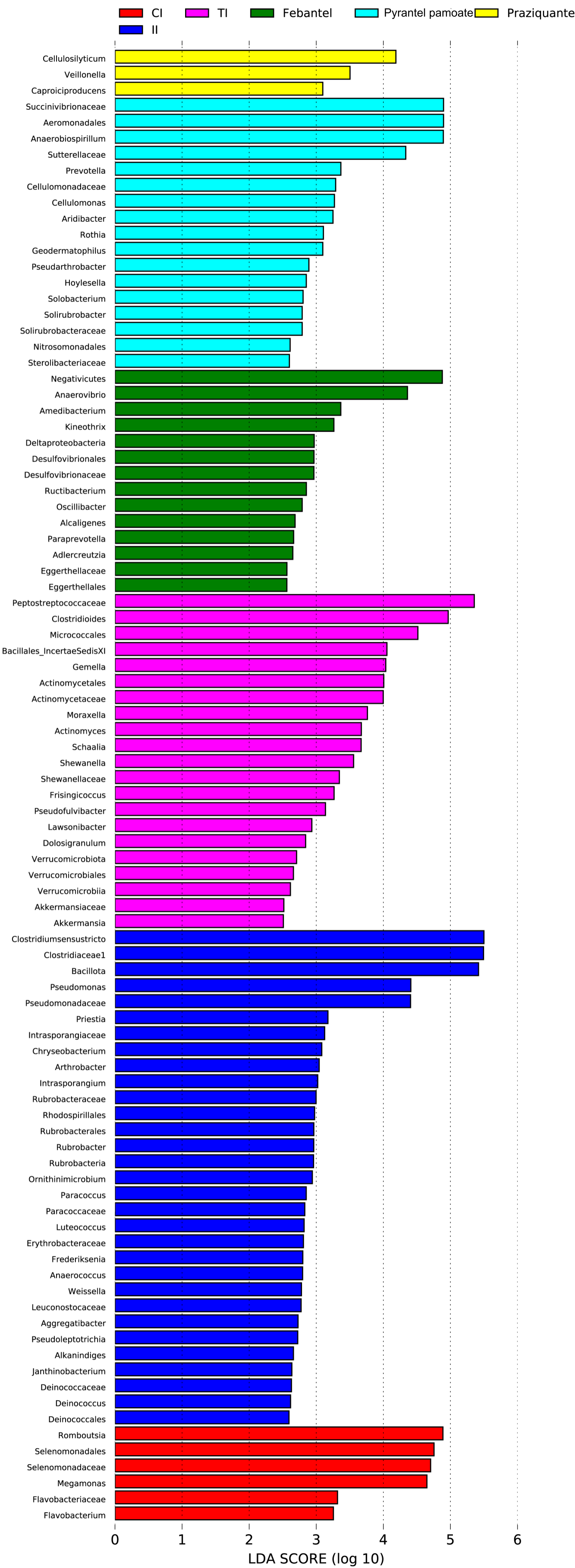

Supplement: Supplementary file 1 [file microorganisms-12-02037-s001.zip › Figure S1.pdf]
